# Mutation in *Irf8* Gene (*Irf8^R294C^*) Impairs Type I IFN-Mediated Antiviral Immune Response by Murine pDCs

**DOI:** 10.3389/fimmu.2021.758190

**Published:** 2021-11-17

**Authors:** Annesa Das, Kuldeep Singh Chauhan, Himanshu Kumar, Prafullakumar Tailor

**Affiliations:** ^1^ Laboratory of Innate Immunity, National Institute of Immunology, New Delhi, India; ^2^ Department of Biological Sciences, Laboratory of Immunology and Infectious Disease Biology, Indian Institute of Science Education and Research (IISER) Bhopal, Bhopal, India; ^3^ Special Centre for Systems Medicine (SCSM), Jawaharlal Nehru University, New Delhi, India

**Keywords:** plasmacytoid dendritic cells (pDCs), type I interferons (IFNs), *IRF8^R294C^*, antiviral immune response, interferon regulatory factor 8 (IRF8), innate immunity, DC activation, interferon stimulated gene (ISG)

## Abstract

Plasmacytoid dendritic cells (pDCs) are the key producers of type I interferons (IFNs), thus playing a central role in initiating antiviral immune response. Besides robust type I IFN production, pDCs also act as antigen presenting cells post immunogenic stimulation. Transcription factor *Irf8* is indispensable for the development of both pDC and cDC1 subset. However, the mechanism underlying the differential regulation by IRF8 in cDC1- and pDC-specific genomic architecture of developmental pathways still remains to be fully elucidated. Previous studies indicated that the *Irf8^R294^* ^C^ mutation specifically abrogates development of cDC1 without affecting that of pDC. In the present study using RNA-seq based approach, we have found that though the point mutation *Irf8^R294^* ^C^ did not affect pDC development, it led to defective type I IFN production, thus resulting in inefficient antiviral response. This observation unraveled the distinctive roles of IRF8 in these two subpopulations—regulating the development of cDC1 whereas modulating the functionality of pDCs without affecting development. We have reported here that *Irf8^R294^* ^C^ mutation also caused defect in production of ISGs as well as defective upregulation of costimulatory molecules in pDCs in response to NDV infection (or CpG stimulation). Through *in vivo* studies, we demonstrated that abrogation of type I IFN production was concomitant with reduced upregulation of costimulatory molecules in pDCs and increased NDV burden in IRF8^R294C^ mice in comparison with wild type, indicating inefficient viral clearance. Further, we have also shown that *Irf8^R294^* ^C^ mutation abolished the activation of type I IFN promoter by IRF8, justifying the low level of type I IFN production. Taken together, our study signifies that the single point mutation in *Irf8*, *Irf8^R294^* ^C^ severely compromised type I IFN-mediated immune response by murine pDCs, thereby causing impairment in antiviral immunity.

## Introduction

Dendritic cells (DCs) are professional antigen-presenting cells that can be subdivided into several classes based on expression of surface markers, transcription factors, and functional specificity (1, 2). DCs are classified broadly into classical dendritic cells (cDCs) and plasmacytoid DCs (pDCs). cDCs are further divided into cDC1 (comprising of splenic CD8α^+^ DCs and tissue resident CD103^+^ DCs) and cDC2 (comprising of splenic CD4^+^ DCs and tissue resident CD11b^+^ DCs) (3, 4). cDC1 plays an important role in antigen cross-presentation, cytokine interleukin 12 (IL12) production, tumor rejection, protection against *Toxoplasma gondii* (5–9), whereas cDC2 has a predominant role in antigen presentation by MHC II, tolerance, cytokine IL6 and IL23 production (1, 10, 11). pDCs are the key players in mounting antiviral responses as they are the major producers of type I interferons (IFNs) upon detecting PAMPs (pathogen-associated molecular patterns) by Toll-like receptor 7 (TLR7) and TLR9. Type I IFN response mediated by pDCs also depends on the virus and the route of infection (12, 13). Type I IFN production subsequently leads to induction of ISGs (interferon stimulated genes), which in turn initiate a series of downstream signaling cascades essential for mounting efficient antiviral immune response (14).

In course of DC development and diversification from a common DC progenitor, the presence of subset specific transcription factors and cytokines in the developmental milieu triggers the lineage-specific developmental program (15–17). *In vitro* bone marrow–derived dendritic cell cultures by employing cytokines such as FLT3L and GM-CSF have aided the studies related to DC development; GMDC (GM-CSF-induced bone marrow–derived DC) cultures give rise to the heterogeneous cell types with similarities to CD11b^+^ cDCs and macrophages, whereas FLDC (FLT3 ligand–induced bone marrow–derived DC) cultures would preferentially develop into pDCs and cDCs (18–23). Study employing *Csf2r* null (*Csf2rb^−/−^Csf2rb2^−/−^*) mice demonstrated significance of GM-CSF signaling in survival and homeostasis of non-lymphoid tissue-resident CD103^+^ and CD11b^+^ DCs (24). Transcription factors *Irf4, Relb, Notch, Klf4*, and different members of NF-κB family play a lead role in cDC2 development (11, 16, 25, 26); whereas *Irf8, Batf3, Id2, Zbtb46, Nfil3, Bcl6* are important for cDC1 development (15, 25, 27–29). pDC development requires the transcription factors *Irf8, Spib, Ikaros, Runx2, E2.2*, etc. (13, 16, 30–33). Earlier studies revealed that transcription factor *Irf8* is essential for development of both cDC1 and pDC (30); however, transcriptional program that leads to generation of these separate DC subpopulations, which vary in morphology and functionality, is still obscure. A previous study reported that *Irf8^R294C^*, a point mutation in *Irf8*, abolishes cDC1 subset development without affecting pDC developmental program (34). During DC lineage diversification, among all the cells expressing IRF8, the balance of *Id2* and *E2.2* decides direction of lineage development. Expression of higher amount of *E2.2* over *Id2* together with *Irf8* shifts developmental regime towards pDC (1, 15, 16, 35, 36). It is intriguing that IRF8^R294C^ mice retain the intact pDC population since previous studies indicated that *Irf8* plays a central role in controlling fate of pDC development and function (30, 34). Recent studies have found that pDCs also develop from lymphoid progenitor (37, 38). But, how *Irf8* plays a role in regulating the divergence of subset specific identity of cDC1 and pDC requires further elucidation. An enhancer element was identified at +32 Kb of *Irf8* which displayed binding of BATF3-IRF8 complex leading to autoactivation of *Irf8* in cDC1 population, suggesting a critical role of IRF8-BATF3 interaction in cDC1 commitment as against the pDC development (39). Recent study describing *Irf8* ^VENUS^ reporter mouse strain having expression of *Irf8* from three intact copies of IRF8 present in the BAC reporter transgenes demonstrated that its over expression could bypass the requirement of *Batf3* for cDC1 development (40). IRF8^R294C^ mice develop spontaneously myeloid leukemia, exhibit splenomegaly, are defective in IL12p40 production, and are more prone to *M. bovis* infection (34, 41, 42). Some of these defects were attributed to cDC1 deficiency; however, further understanding of the mechanism of action and the effect of the mutation *Irf8^R294C^* especially in pDC subset in mounting immune responses will provide valuable insights in pDC biology. In depth analysis of DC subset development resulting from *Itgax-cre* mediated deletion of *Irf8* expression revealed that it did not affect pDC development or maintenance of fully developed pDCs but caused a major shift in their function (43). In the present study, to better understand the impact of *Irf8^R294C^* mutation in the pDC subset, using RNA-seq approach we compared the splenic pDC transcriptome of IRF8^R294C^ with IRF8^WT^ (wild type) mice for the first time. We report that *Ifnb1* gene (encoding IFN-β) is downregulated in pDCs from IRF8^R294C^ mice. This observation is of great significance since pDC is characterized as the major type I IFN-producing cells, and dysregulation of pDCs has been associated with several autoimmune diseases such as psoriasis, systemic lupus erythematosus (SLE), rheumatoid arthritis, etc. (44–48). Further, the role of pDCs is also recognized in viral infection such as hepatitis B as pDC impairment is observed in chronic HBV patients (49).

It was reported that IRF8^R291Q^ mutation in human, orthologous to murine IRF8^R294C^ mutation, results in several immunological consequences (50), suggesting the importance of this point mutation regarding functioning of immune system. So, understanding the pDC development and function in context of *Irf8^R294C^* mutation can be of utmost importance to diagnostics and in developing the course of therapeutics. Here, using NDV (Newcastle disease virus) infection and CpG stimulation model, we have demonstrated that *Irf8^R294C^* mutation leads to impairment in type I IFN production. In addition to this, we have determined that the impairment in type I IFN production aggravates the defect in production of ISGs and upregulation of costimulatory molecules in pDCs from IRF8^R294C^ mice. Further, using IRF8^R294C^ mice we have shown that under *in vivo* experimental condition also, IRF8^R294C^ mice are defective in type I IFN production, upregulation of costimulatory molecules in pDC, and exhibit increased NDV burden. Collectively, this study contributes to better understanding of the novel role of *Irf8^R294C^* mutation in pDC functionality especially in interferon production and antiviral response. These findings unravel differential role of *Irf8* due to point mutation *Irf8^R294C^* in pDC function, casting importance in therapeutic implications.

## Materials and Methods

### Mice and Cell Culture

All animal experiments were conformed to the guidelines of the animal ethics committee at National Institute of Immunology (NII) (IAEC approval# 536/19) and biosafety committee guidelines (IBSC approval# 413/20). IRF8^−/−^ and IRF8^R294C^ mice were procured from Jackson Laboratory. IRF8^R294C^ mice were crossed with C57BL/6 wild-type female for at least three generations to eliminate the vertical transmission of Murine Leukemia Virus (MuLV). Progenies were crossed to generate the mice carrying homozygous alleles for the mutation, and all the mice were used at the age of 8–12 weeks. Bone marrow mononuclear cells were isolated from the femur and tibia and cultured in the presence of FLT3L (100 ng/ml, PeproTech) as described previously (51). After day 8, loosely adherent cells were harvested and used for different treatments, stimulations, and experiments. For stimulation with CpG (1826 ODN, Merck), as employed earlier (23, 27, 43), cells were stimulated with 1 μg/ml CpG for the mentioned time period and were analyzed for transcript or proceeded to flow cytometry. For the 7-AAD experiment, cells were harvested after mentioned time point and stained with different fluorochrome conjugated antibodies and finally with 7-AAD before analyzing through flow cytometry. NDV (LaSota strain) was a kind gift from Dr. Himanshu Kumar (IISER Bhopal). NDV infections were set up in serum-free media for 90 min at MOI 1 followed by replenishment with fresh culture media for the mentioned time period after washing with PBS. DCs were stained for surface markers with the following immunophenotyping antibodies: anti-B220 FITC (RA3-6B2), anti-CD11c APC (N418), anti-CD24 PE (M1/69), anti-CD80 V450 (16-10A1), anti-CD8α PE (53-6.7), anti-CD4 APC Cy7 (GK1.5), anti-Sirpα BV421 (P84) (all from BD biosciences); anti-CD40 FITC (HM40-3) (from eBioscience); anti-CD86 PE (GL-1) (from BioLegend), and biotin-labeled anti-SiglecH (551.3D3) (Miltenyi Biotech). Events were captured in FACS CantoII using FACS Diva software, and data were analyzed using Flowjo software (version 9.3.2).

HEK293T and U2OS cells were maintained in DMEM supplemented with 10% FBS, 100 U/ml penicillin, and 50 μg/ml streptomycin. Retroviral vectors expressing murine IRF8^WT^ (wild type) and IRF8^R294C^ were generated, and HEK293T cells were seeded in six-well dish for retroviral transduction with viral supernatants and 4 μg/ml polybrene by spinoculation as described previously (52). Transduced cells were then used for further experiments.

### RNA Sequencing

Spleens were harvested from 8- to 12-week-old mice, and splenocytes were isolated. Briefly, dendritic cell-enriched, low-density cell fraction from spleen digested by Liberase (Roche laboratory) was isolated using 30% BSA in PBS as described previously (25). The isolated cells were stained using anti-CD11c, anti-B220, anti-CD8α, anti-CD4 (all from BD biosciences), and biotin-labeled anti-SiglecH (Miltenyi Biotech) antibodies; and CD11c^+^B220^+^SiglecH^+^ pDCs were sorted using FACS Aria III cell sorter. Sorted cells were lysed in RNAiso Plus (Takara), and RNA was isolated using RNeasy mini kit (Qiagen). After the QC procedures, mRNA was enriched using oligo(dT) beads. Then, the mRNA was fragmented randomly by adding fragmentation buffer, and cDNA was synthesized using mRNA template and random hexamer primers, after which a custom second-strand synthesis buffer (Illumina), dNTPs, RNaseH, and DNA polymerase I were added to initiate the second-strand synthesis. Followed by terminal repair, A ligation, and sequencing adaptor ligation, the double-stranded cDNA library was completed through size selection and PCR enrichment. Finally, RNA sequencing was carried out in Illumina platform (150 bp paired end), and generated paired end reads were used for downstream analysis. Raw data QC was carried out to check data quality. The quality trimmed reads were aligned using HISAT2 aligner (v.2.1.0) and mapped to GRCm39 as reference genome. HTSeq-count (v.0.12.4) was utilized to generate read count, and DESeq2 (v.1.30.1) R-package was employed for differential gene expression analysis.

### RNA Extraction and qRT-PCR

RNA was isolated using Nucleospin RNA isolation kit (Machery-Nagel), and cDNA was prepared using high-capacity reverse transcription kit (Applied Biosystems) according to the manufacturer’s protocol. cDNA was diluted appropriately, and qRT-PCR was performed using SYBR Green PCR master mix and 7500 Fast RT PCR machine (Applied Biosystems). Relative mRNA expression was calculated using delta Ct method, and to normalize the variability in expression level, *Gapdh* was used as internal control. Primers used for qRT-PCR are listed in **Supplementary Table 1**.

### Dual Luciferase Reporter Assay

U2OS cells seeded in 24-well plates were co-transfected with 100 ng of reporter plasmid pGL4.20-Luc (Blank), pGL4.20-Ifnβ-Luc containing promoter region of murine *Ifnb* promoter alone (control), pGL4.20-Ifnα6-Luc containing promoter region of murine *Ifna6* promoter alone (control) or along with pcDNA-IRF8^WT^ or IRF8^R294C^ (300ng) expressing plasmids. Transfection was carried out using Lipofectamine 2000 according to the manufacturer’s protocol and as transfection control *Renilla* luciferase plasmid pRL-TK (Promega, 50 ng) was used. Cells were harvested 24 h post-transfection, and protein activity of firefly and *Renilla* luciferase was quantified using dual-luciferase reagents (Promega) and Sirius Luminometer V3.2. *Renilla* luciferase was used to normalize firefly luciferase activity.

### *In Vivo* NDV Infection Experiment

NDV stock was prepared in embryonated chicken eggs and titrated by hemagglutination assay as described previously (53) in Dr. Himanshu Kumar’s laboratory in IISER Bhopal. Then 1,200 hemagglutination assay (HA) unit of NDV was used for the infection where HA unit denotes the HA titer of the virus calculated as reciprocal of highest dilution at which complete agglutination of RBC was observed in hemagglutination assay. Eight- to 12-week-old mice were injected with PBS (control) or 1,200 HA NDV intravenously and kept for 8 h. Then blood was collected and spleens were harvested. DC-enriched low-density cell fraction from spleen was isolated as mentioned above. Blood was centrifuged at 2,000 g for 15 min, and serum was collected and stored for further analysis.

### ELISA

FLDC culture supernatants after 24 h treatment or serum from *in vivo* experiment were subjected to IFN-α1 and IFN-β ELISA using Abcam IFN-α1 (ab252352) and Abcam IFN-β (ab252363) ELISA kits respectively according to the manufacturer’s protocol.

### Statistical Analysis

Statistical analysis was performed using unpaired Student’s t test, and significance is represented as *p < 0.05, **p < 0.01, ***p < 0.001, and ns means non-significant.

## Results

### *Irf8^R294C^* Mutation Alters the Transcriptome of pDC Subset

Numerous studies established that *Irf8* is a key transcription factor for cDC1 and pDC development (1, 25, 30, 54). The characterization of splenic DCs and FLDC cultures from *Irf8^−/−^* mice exhibited complete absence of both cDC1 and pDC populations; however, *Irf8^R294C^* mutation selectively abrogated cDC1 development, without affecting that of pDC (**Supplementary Figures 1A, B**) in coherence with previous studies (30, 34, 54). This intriguing phenomenon of selective loss of cDC1 population in IRF8^R294C^ but retention of pDC population led us to investigate whether *Irf8* plays a differential role in cDC1 and pDC development and function. We found that transcript level of *Irf8* is elevated in pDCs from IRF8^R294C^ mice in comparison with IRF8^WT^ (wild type) mice (**Supplementary Figure 1C**). The increased level of *Irf8* transcript in IRF8^R294C^ pDCs led us to further explore the role of IRF8 in pDC biology.

**Figure 1:**
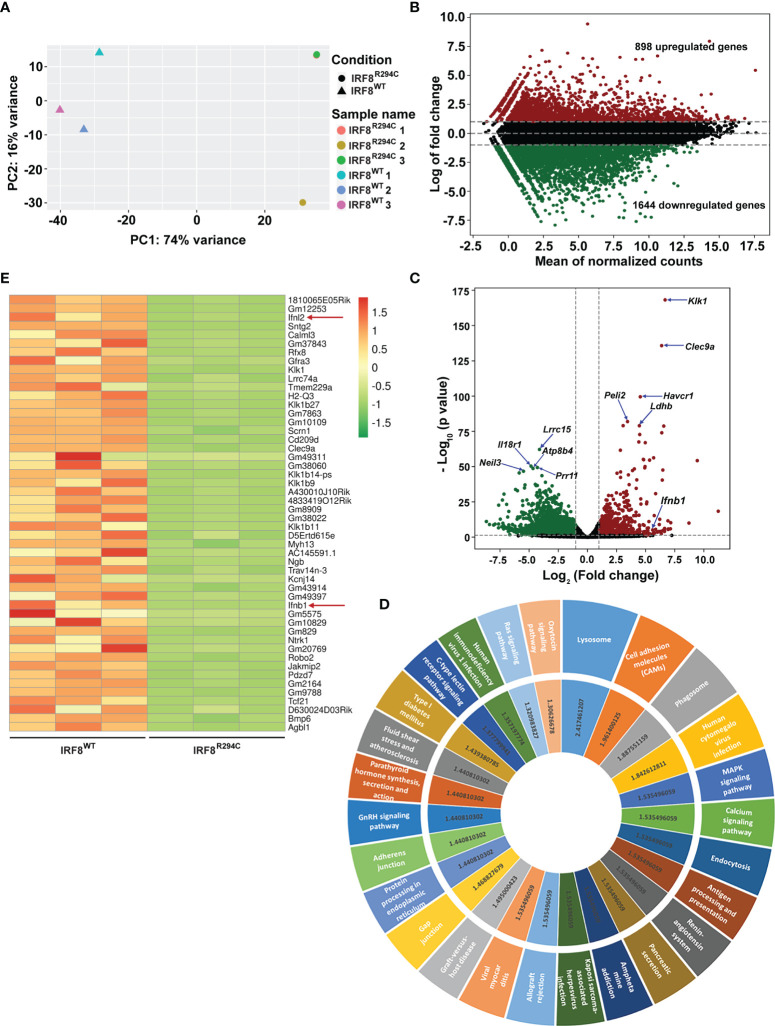
*Irf8^R294C^* mutation leads to alteration in pDC transcriptome. **(A)** Plot showing principal component analysis of six samples; distance between the samples depicts the difference in gene expression among the two sets (IRF8^R294C^ samples 1 and 3 are overlapping, hence appearing as a single dot). **(B)** MA plot presenting differential gene expression, indicating upregulated (in red) and downregulated (in green) genes in IRF8^WT^. **(C)** Volcano plot displaying the differential gene expression between the two groups of samples with upregulated and downregulated genes in IRF8^WT^ illustrated in red and green, respectively. **(D)** KEGG pathway analysis of upregulated genes in IRF8^WT^; the outer circle represents top upregulated pathways, and the inner circle indicates corresponding –log_10_FDR value of respective KEGG pathway. **(E)** Heatmap depicting relative expression of top 50 genes across six samples with red and green color denoting upregulated and downregulated genes in IRF8^WT^, respectively.

To investigate this, we sorted pDCs (CD11c^+^ B220^+^ SiglecH^+^) from IRF8^WT^ and IRF8^R294C^ mice spleen, carried out transcriptome analysis, and studied differentially expressed genes (**Supplementary Figure 2A**). Principal component analysis (PCA) of RNA-seq data showed differential segregation of clusters of IRF8^WT^ and IRF8^R294C^ pDCs (**Figure 1A**). Additional analysis demonstrated a total of 3,494 genes were differentially expressed between these two groups after adjusting the p value cutoff <0.05. Further setting the next cutoff parameter of Log_2_FC value implemented at <=−1 or >=1, the number of differentially regulated genes were reduced to 2,542. Out of 2,542 genes, 898 genes were found to be upregulated in IRF8^WT^ and the rest of the genes (1,644) were upregulated in IRF8^R294C^. To visualize gene expression data sets, the two-dimensional (2D) scatter MA plot (Bland Altman plot, “M” denotes log ratio and “A” denotes mean average) was constructed (**Figure 1B**). Global gene expression values, which were obtained from analysis of IRF8^WT^ and IRF8^R294C^ transcriptomic data, were represented in volcano plot (**Figure 1C**).

To further understand the ontological features of differentially regulated genes, we implemented KEGG pathway analysis on divergently upregulated (**Figure 1D**) and downregulated (**Supplementary Figure 2B**) genes in IRF8^WT^ pDCs. Genes significantly enriched in KEGG pathway analysis included those involved in different pathways related to immune response and viral infections such as antigen processing and presentation, human cytomegalovirus infection, human immunodeficiency virus 1 infection, etc., and genes regulating essential signaling mechanisms such as MAPK signaling pathway, calcium signaling pathway, Ras signaling pathway, etc. Genes that showed differential expression in IRF8^WT^ and IRF8^R294C^ pDCs were represented in heatmaps (**Supplementary Figures 2C, D** and **Figure 1E**). Intriguingly, among various genes, the gene *Ifnb1* (also known as *Ifnb*), which encodes interferon β (IFN-β), was found to be significantly downregulated in pDCs from IRF8^R294C^ than IRF8^WT^ mice (**Figures 1C, E**). IFN-β, a key cytokine belonging to type I IFN family, plays pivotal role in pDC biology and is well established for its indispensability in mediating antiviral immune response (12, 13). Also, *Ifnl2*, which encodes interferon lambda 2 (also known as IL28A), was found to be downregulated in IRF8^R294C^ (**Figure 1E**). IFNL2, distantly related to type I IFN and IL10 family, is important for host defense from antiviral challenge and is involved in antitumor activity (55–58). From these analyses, we can clearly infer that although the point mutation *Irf8^R294C^* does not affect development of pDC subset, it results in significant changes at the transcriptome level.

Since *Irf8* is a fundamental transcription factor involved in DC development and the fact that pDCs, which are the key producers of type I IFN, are showing decreased level of *Ifnb* in IRF8^R294C^ mice strongly suggest the probable impact of this specific point mutation in changing the pDC transcriptome and immune response. So, in IRF8^R294C^ pDCs, the decreased expression of *Ifnb* further prompted us to investigate the effect of *Irf8^R294C^* mutation in immune response to gain mechanistic insight of the expression and regulation of downstream signaling molecules.

### IRF8^R294C^ pDCs Are Defective in Type I IFN Production

**Figure 2:**
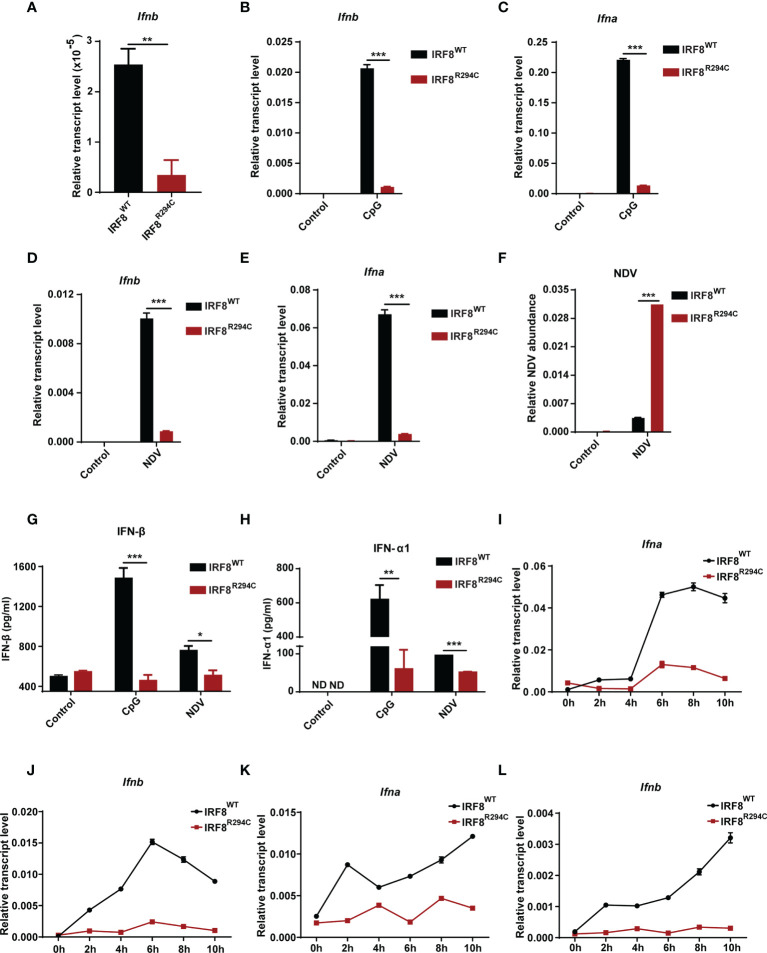
IRF8^R294C^ pDCs show abrogation of type I IFN production *in vitro*. **(A)** RNA was isolated from IRF8^WT^ and IRF8^R294C^ FLDCs and analyzed by qRT-PCR to measure *Ifnb* expression. FLDC cultures from IRF8^WT^ and IRF8^R294C^ mice were stimulated with CpG (1 μg/ml) for 8 h, and level of *Ifnb* **(B)** and *Ifna* **(C)** transcript expression was checked by qRT-PCR. FLDC cultures from IRF8^WT^ and IRF8^R294C^ mice infected with NDV for 8 h were checked for expression of *Ifnb* **(D)**, *Ifna* **(E)** transcripts, and NDV abundance **(F)** by qRT-PCR. FLDC culture supernatants from IRF8^WT^ and IRF8^R294C^ mice stimulated with CpG or infected with NDV for 24 h were examined for expression of IFN-β **(G)**, IFN-α1 **(H)** cytokine by ELISA. FLDC cultures from IRF8^WT^ and IRF8^R294C^ mice stimulated with CpG (1 μg/ml) for indicated time points were analyzed for *Ifna* **(I)** and *Ifnb* **(J)** transcripts by qRT-PCR. FLDC cultures from IRF8^WT^ and IRF8^R294C^ mice infected with NDV for indicated time points were analyzed for *Ifna* **(K)** and *Ifnb* **(L)** transcripts by qRT-PCR. Data **(A–F)** are representative of three independent experiments with error bar representing + standard error of mean (SEM). Data **(G, H)** are mean of three independent experiments with error bar representing + standard error of mean (SEM). Data **(I–L)** are representative of three independent experiments with error bar representing ± standard error of mean (SEM). ND, not detected; *p < 0.05, **p < 0.01, ***p < 0.001; p value obtained from Student’s t test.

First, to confirm observed reduction of *Ifnb* expression in RNA-seq data, we determined the transcript level of *Ifnb* from FLDC cultures derived from IRF8^WT^ and IRF8^R294C^ mice by qRT-PCR and found that indeed *Ifnb* was downregulated in IRF8^R294C^ compared to its wild-type counterpart (**Figure 2A**). Next, we wanted to examine the impact of *Irf8^R294C^* mutation on type I IFN production upon CpG stimulation (TLR9 agonist) in FLDC cultures as pDCs induce type I IFN transcription upon encountering CpG by TLR9. It was found that induction of both *Ifnb* and *Ifna* (type I IFN) transcripts were greatly reduced in IRF8^R294C^ DCs post-stimulation (**Figures 2B, C**). As pDCs play a major role in initiating antiviral response through type I IFN production, we wanted to determine the type I IFN production upon viral infection. To address this, FLDC cultures from IRF8^WT^ and IRF8^R294C^ mice were infected by a single-stranded RNA virus—Newcastle disease virus (NDV). NDV infection in IRF8^WT^ led to marked augmentation of type I IFN transcript level, whereas in IRF8^R294C^ there was abrogation of type I IFN expression (**Figures 2D, E**), which was consistent with the result from CpG stimulation.

Further, defective induction of type I IFN transcripts in IRF8^R294C^ FLDCs was concomitant with increased NDV burden in comparison with IRF8^WT^ (**Figure 2F**). This observation was further confirmed in the protein level by ELISA as we observed IFN-β and IFN-α1 were greatly declined in FLDC culture supernatants from IRF8^R294C^ mice compared to IRF8^WT^ upon CpG stimulation as well as NDV infection (**Figures 2G, H**). So, these experiments infer that the pDCs from IRF8^R294C^ mice are defective in type I IFN production. Further, we wanted to ascertain the effect of this mutation on the time kinetics of type I IFN production upon immune challenge. FLDCs from IRF8^R294C^ mice stimulated with CpG were found to be defective in type I IFN production starting from early time points to late time points (**Figures 2I, J**). Next, we examined time kinetics of type I IFN production in FLDCs infected with NDV and found that in this scenario also, type I IFN production was greatly hindered in IRF8^R294C^ in comparison with IRF8^WT^ (**Figures 2K, L**). In summary, these data conclude that IRF8^R294C^ pDCs are defective in type I IFN production from very early time points to late time points.

**Figure 3:**
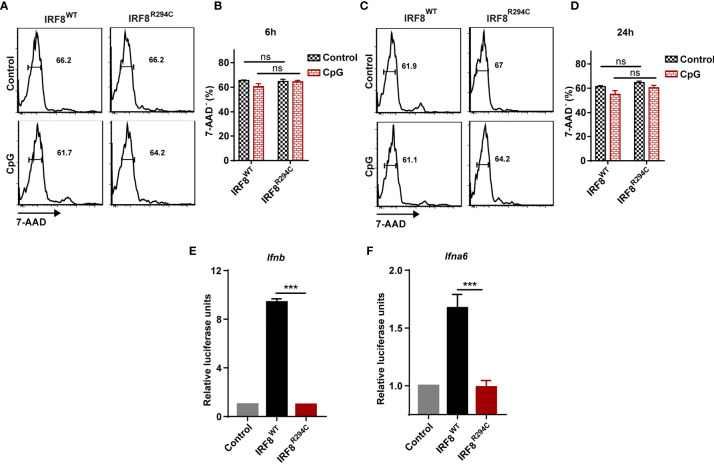
Defect of type I IFN production by IRF8^R294C^ pDCs is not due to increased cell death. FLDC cultures from IRF8^WT^ and IRF8^R294C^ mice were kept unstimulated or stimulated with CpG and stained for different surface markers followed by 7-AAD staining. Cells with negative staining for 7-AAD were considered as live cells. Flow cytometric analysis **(A)** and quantitation **(B)** of live CD11c^+^ B220^+^ SiglecH^+^ pDCs from IRF8^WT^ and IRF8^R294C^ FLDC cultures after 6 h time point. Flow cytometric analysis **(C)** and quantitation **(D)** of live CD11c^+^ B220^+^ SiglecH^+^ pDCs from IRF8^WT^ and IRF8^R294C^ FLDC cultures after 24 h time point. IRF8^WT^ activates expression of luciferase reporter construct driven by *Ifnb* **(E)** and *Ifna6* **(F)** promoters, while IRF8^R294C^ was unable to induce the expression of promoters. Data **(A, C)** are representative of three independent experiments. Data **(B, D, E, F)** are mean of three independent experiments with error bar representing + SEM and ***p < 0.001, ns, non-significant. p value obtained from Student’s t test.

We speculated that the defect of type I IFN production in IRF8^R294C^ FLDCs may be explained by either increased cell death in IRF8^R294C^ or due to defect in pDC transcriptional program regulated by IRF8 or any other plausible mechanism mediated by the point mutation *Irf8^R294C^*. To examine if the decreased production of type I IFN was due to increased cell death in IRF8^R294C^, we stimulated FLDCs derived from IRF8^WT^ and IRF8^R294C^ mice with CpG for 6 h and 24 h and stained the treated as well as untreated cells with viability dye 7-AAD. Flow cytometric analysis revealed that the live pDC percentages were similar in IRF8^R294C^ with that of IRF8^WT^ mice under untreated as well as CpG stimulated condition for both 6 h and 24 h time points (**Figures 3A–D**). This experiment clearly refuted the hypothesis that reduced type I IFN production in IRF8^R294C^ was due to increased cell death.

Next, we wanted to decipher if this impairment of type I IFN production in IRF8^R294C^ pDCs was an effect of indirect regulation of other pDC-specific factors or due to any mechanism mediated by mutation *Irf8^R294C^* itself. RIG-I like receptor (RLR) is a family of cytosolic pathogen recognition receptor (PRR) which recognizes viral RNA and induces type I IFN production (59). Fibroblasts are known to mediate antiviral response through RLR signaling (60), and mechanism for sensing NDV in HEK293T by RLR differs from that of pDCs mediated by TLR. HEK293T cell line was employed previously for studying interferon induction in response to viral infection (61). Hence, to further investigate, we infected HEK293T cells stably expressing IRF8^WT^, IRF8^R294C^ separately with NDV, studied time kinetics of type I IFN transcript level (**Supplementary Figures 3A, B**) and found that type I IFN induction was curtailed in IRF8^R294C^-expressing cells. This experiment indicated that point mutation *Irf8^R294C^* directly affects the type I IFN production.

### Defect in Type I IFN Production by IRF8^R294C^ pDCs Leads to Defective Induction of ISGs

**Figure 4:**
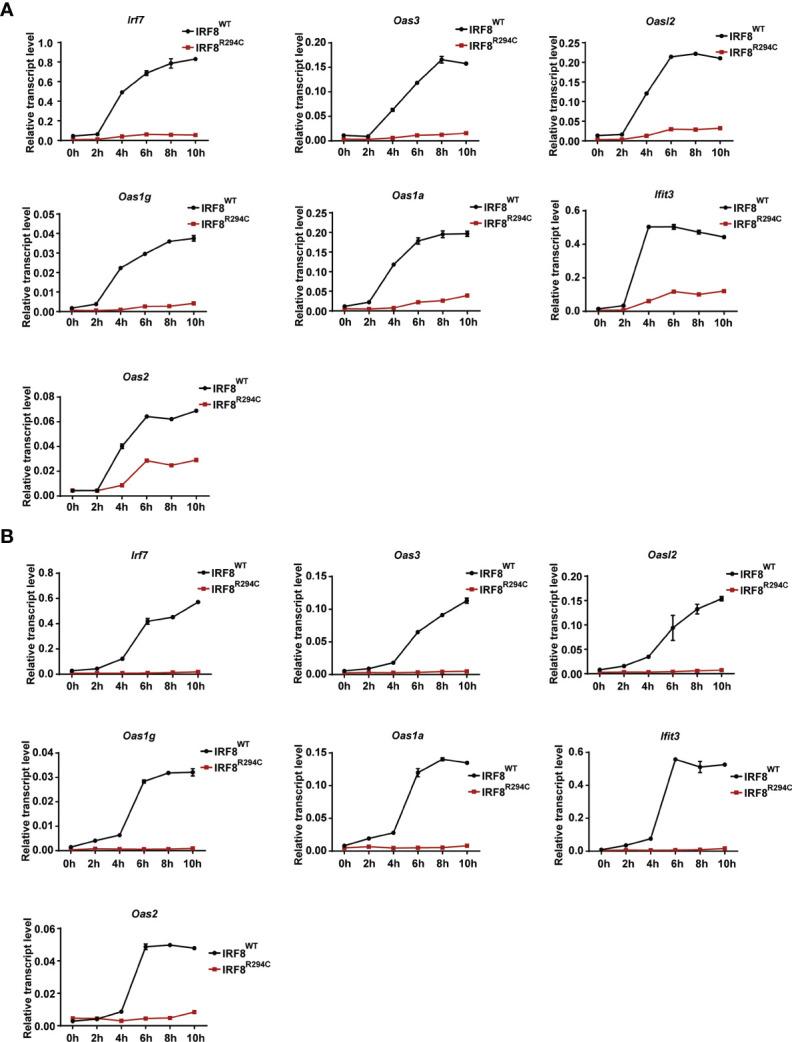
Impaired type I IFN production in IRF8^R294C^ pDCs leads to impairment in induction of ISGs. Transcript analysis of *Irf7, Oas3, Oasl2, Oas1g, Oas1a, Ifit3*, and *Oas2* by qRT-PCR in FLDC cultures from IRF8^WT^ and IRF8^R294C^ mice stimulated with CpG **(A)** or infected with NDV **(B)** at indicated time points. Data **(A, B)** are representative of three independent experiments with error bar representing ± SEM.

Next, we wanted to investigate the mechanism and the downstream effect of impaired type I IFN production. We hypothesized that the inability of type I IFN production in IRF8^R294C^ could be due to ineffective type I IFN promoter activation. Through promoter assay we demonstrated that, IRF8^WT^ induced *Ifnb* and *Ifna6* promoters, but induction of *Ifnb* and *Ifna6* promoters was defective in IRF8^R294C^-expressing cells (**Figures 3E, F**). This observation suggested that inefficient promoter activation by IRF8^R294C^ translated to abrogated type I IFN production. Upon generation, type I IFN further promotes transcription of other effector molecules of several downstream signaling cascades which carry out different aspects of immune response (14). So, we speculated the defective type I IFN production may result in ineffective downstream signaling subsequently. We observed that, indeed, the production of ISGs was declined significantly in FLDCs from IRF8^R294C^ mice stimulated with CpG compared to IRF8^WT^ (**Figure 4A**). Similar observations were made in FLDCs from IRF8^R294C^ mice infected with NDV (**Figure 4B**). IRF7 plays an important role in type I IFN-mediated signaling pathway and is known to be one of the major regulators of the pathway (59). IFIT3 and other ISGs—OAS3, OAS2, OAS1a, OAS1g, OASL2—which are 2’-5’ oligoadenylate synthetases, are involved in mounting antiviral innate immune response by restricting viral replication (62–64). Overall, these results suggest that the reduced expression of ISGs reflects the inability in induction of type I IFNs, and reduced production of these ISGs due to impaired type I IFN production in IRF8^R294C^ may further compromise the response to CpG stimulation and NDV infection.

**Figure 5:**
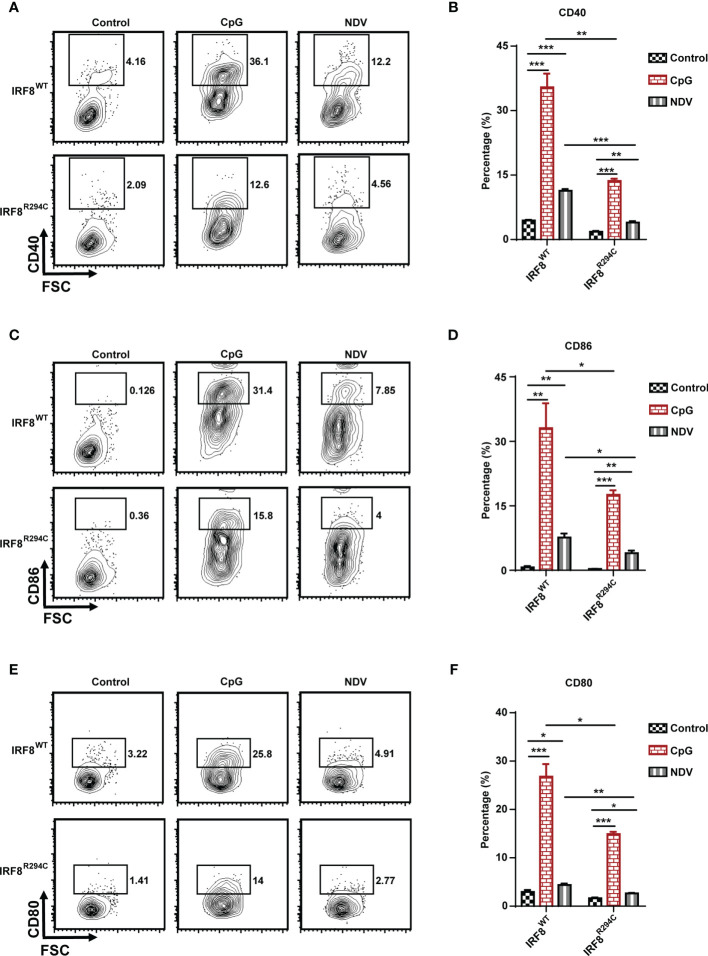
IRF8^R294C^ pDCs are defective in activation upon immune challenges. FLDCs from IRF8^WT^ and IRF8^R294C^ mice were kept unstimulated or stimulated with CpG or infected with NDV, and CD11c^+^SiglecH^+^ pDCs were analyzed for expression of costimulatory molecules. Flow cytometric analysis of expression of **(A)** CD40, **(C)** CD86, **(E)** CD80 markers, and quantitation of expression of the respective markers **(B**, **D**, **F)** are shown. Data **(A, C, E)** are representative of three independent experiments. Data **(B, D, F)** are mean of three independent experiments with error bar representing + SEM and *p < 0.05, **p < 0.01, ***p < 0.001. p value obtained from Student’s t test.

pDCs in resting state express the costimulatory molecules in low level, but once pDCs are activated, they upregulate several costimulatory molecules (CD40, CD86, CD80) and initiate a cascade of activities leading to instigating adaptive immune response (13, 65, 66). So, to characterize pDC activation, we next assessed the upregulation of costimulatory molecules in pDCs from IRF8^WT^ and IRF8^R294C^ mice stimulated with CpG or infected with NDV. In comparison to IRF8^WT^, the IRF8^R294C^ pDCs displayed significant partial but not complete reduction in upregulation of CD40, CD86, and CD80 expression upon CpG stimulation as well as NDV infection (**Figures 5A–F**). Taken together, these experiments suggest that *Irf8^R294C^* point mutation renders pDCs defective in activation as the consequence of impaired type I IFN induction, leading to deficient expression of costimulatory molecules.

### *In Vivo* Study Confirms the Defective Type I IFN Production in IRF8^R294C^ Mice

**Figure 6:**
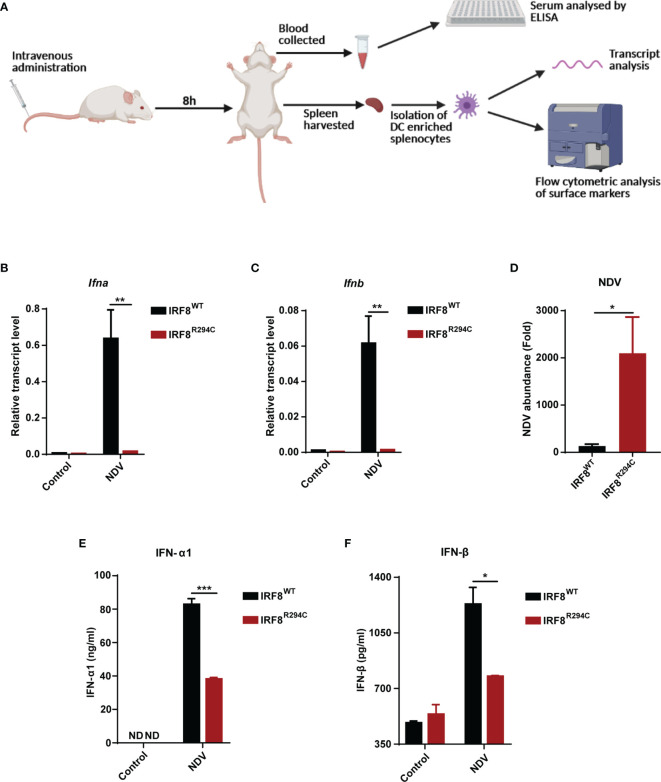
Impairment of type I IFN production in IRF8^R294C^ mice upon NDV infection. **(A)** Schematic representation of the workflow for *in vivo* NDV infection. Transcript analysis of *Ifna* **(B)**, *Ifnb* **(C),** and abundance of NDV **(D)** in splenocytes from uninfected or NDV-infected IRF8^WT^ and IRF8^R294C^ mice by qRT-PCR. IFN-α1 **(E)** and IFN-β **(F)** concentration in sera from uninfected or NDV-infected IRF8^WT^ and IRF8^R294C^ mice were quantitated by ELISA. Data **(B–D)** shown are mean value with error bar representing + SEM (n= 6 mice per group). Data **(E, F)** shown are mean value with error bar representing + SEM (n= 3 mice per group) and *p < 0.05, **p < 0.01, ***p < 0.001; p value obtained from Student’s t test.

**Figure 7:**
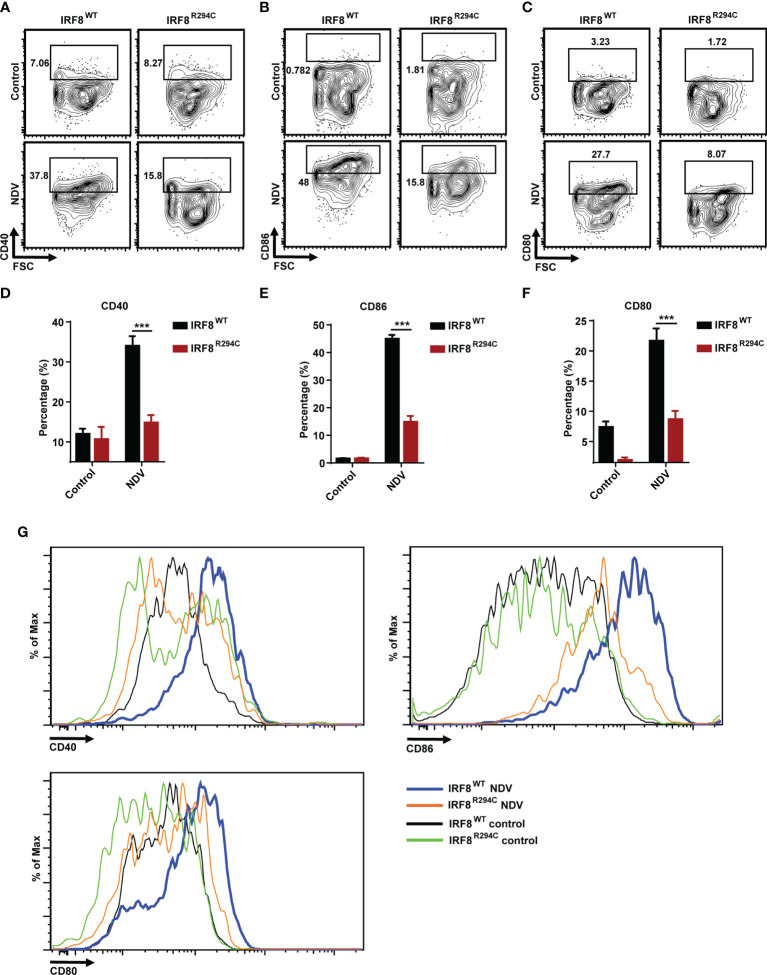
IRF8^R294C^ mice upon NDV infection are inefficient in pDC activation. Mice infected in 6(A) were checked for status of costimulatory molecules among splenic CD11c^+^SiglecH^+^ pDCs. Flow cytometric analysis of surface markers CD40 **(A)**, CD86 **(B)**, CD80 **(C)**, and quantitation of expression of the respective markers **(D–F)** and histogram **(G)** are shown. Data **(D–F)** shown are mean value with error bar representing + SEM and ***p < 0.001 (n= 6 mice per group). Data **(A–C, G)** shown are representative of each group (n= 6 mice per group). p value obtained from Student’s t test.

To corroborate our *in vitro* studies, we infected both IRF8^WT^ and IRF8^R294C^ mice with NDV (**Figure 6A**) and investigated type I IFN induction, NDV transcript, and status of costimulatory molecules in spleen, as spleen is the major site to elicit successful antiviral immune response against systemic infection (67, 68). There was significant impairment in production of type I IFN transcripts in NDV-infected IRF8^R294C^ mice splenocytes compared to IRF8^WT^ counterpart (**Figures 6B, C**). Further, the absence of type I IFN transcript augmentation was associated with increased viral burden in IRF8^R294C^ mice splenocytes (**Figure 6D**). Next, we examined the concentration of IFN-α1 and IFN-β in sera by ELISA. Consistent with increased transcript levels, the upregulation of serum IFN-α1 and IFN-β in IRF8^WT^ mice infected with NDV but not IRF8^R294C^ counterpart bolstered that our observation from *in vitro* system indeed translated to *in vivo* infection condition (**Figures 6E, F**). Next, we examined the activation status of splenic pDCs from infected and uninfected mice. Flow cytometric analysis of splenic pDCs further revealed that IRF8^WT^ mice infected with NDV showed significant upregulation of all the costimulatory molecules—CD40, CD86, CD80 (**Figures 7A–F**). In IRF8^R294C^ mice, upregulation of CD40, CD86, and CD80 in pDCs was compromised upon NDV infection. Also, IRF8^R294C^ pDCs, in unstimulated condition, showed lower expression of CD80 than wild-type counterpart in contrast to previous study in *Irf8^fl/fl^Itgax-cre* mice (43), suggesting the difference in IRF8^R294C^ pDCs carrying mutant IRF8 as against its deletion in *Irf8^fl/fl^Itgax-cre* counterpart. We also compared the MFI of these costimulatory molecules to study the average expression of these markers on each cell (**Supplementary Figures 4A–C**). Expression of CD40, CD80, and CD86 displayed clear shift in histogram of respective markers in IRF8^WT^ mice infected with NDV than all the other experimental groups (**Figure 7G**). Collectively, these experiments indicate that decreased type I IFN production in IRF8^R294C^ mice further leads to immune suppression.

**Figure 8:**
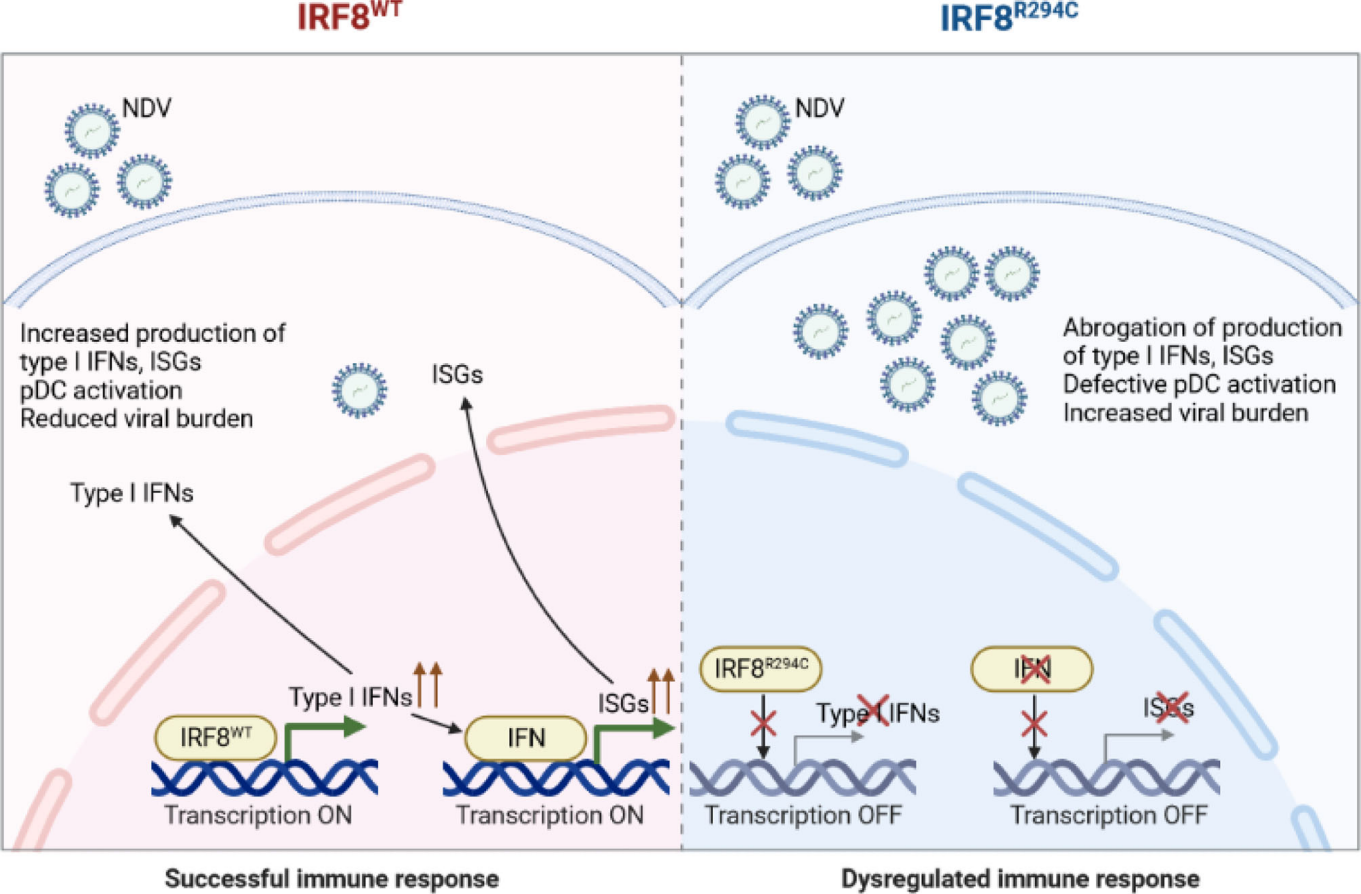
Schematic model of *Irf8^R294C^*-mediated impairment in immune response. Upon NDV infection, IRF8^WT^ induces type I IFN promoter and transcription initiates. Type I IFN further results in production of downstream ISGs and pDC activation. Thus, together, type I IFN and ISGs restrict viral replication and mount successful immune response. Whereas, IRF8^R294C^ fails to induce type I IFN promoter, and transcription does not take place. So, the absence of type I IFN leads to impaired pDC activation and abrogation of production of ISGs concomitant with increased NDV burden. Thus, IRF8^R294C^ results in dysregulated immune response.

Conclusively, these results infer that the inability of mutant IRF8^R294C^ to induce type I IFN promoters translates to impaired type I IFN production, which further abrogates production of ISGs and impairs pDC activation, ultimately leading to increased viral burden and futile immune response (**Figure 8**). Taken together, our study unravels the important role of IRF8 in mediating a successful immune response by controlling pDC function and the differential role played by IRF8 with respect to the cDC1 and pDC subsets. While in cDC1 subset, IRF8 controls the development and differentiation, in pDC subset, IRF8 controls the functionality, and *Irf8^R294^* ^C^ mutation greatly changes the functions mediated by IRF8.

## Discussion

*Irf8* is an important member of IRF family that plays a critical role in regulation of DC differentiation (30). Identification of *Irf8^R294C^* point mutation emphasized the requirement of subsequent studies regarding the role of IRF8 in DC lineage differentiation program, as IRF8^R294C^ mutation results in obliteration of only cDC1 subset but not pDC population in spite of the previous notion of the importance of IRF8 in pDC development from mice knockout studies (30, 34). To delineate the role of IRF8 in DC development and function, in this study we carried out the RNA-seq analysis to characterize the transcriptome of pDCs derived from IRF8^WT^ and IRF8^R294C^ mice. Our analysis reveals that *Ifnb* is downregulated in IRF8^R294C^ pDCs than IRF8^WT^ counterpart. pDCs, demonstrating plasma cell–like morphology, secrete massive amounts of type I IFN upon pathogen recognition through TLR7 and TLR9. This extraordinary feature of robust type I IFN production by pDCs further leads to initiation of the downstream signaling to mount host defense against various pathogens (13, 14). pDCs are documented to induce IFN production in response to a wide range of microbial pathogens such as dengue virus, influenza virus, *Toxoplasma gondii*, etc. (69–71). Recent report suggests that pDC frequency was decreased in PBMCs from individuals infected with SARS-CoV-2 and also, in response to TLR stimuli, pDCs from infected individuals produced decreased amounts of IFN-α (72). These observations indicate the important role of type I IFN response, controlled by pDC, against several immune challenges. Due to this essentiality of type I IFN signaling in pDC biology, decreased basal level of *Ifnb* in IRF8^R294C^ pDCs is significant to the field and provides some cognizance in the immune-deficient nature of IRF8^R294C^ mice that was previously reported (42).

Our subsequent experiments, which illustrate the drastic hindrance in type I IFN production in IRF8^R294C^ FLDCs upon NDV infection (or CpG mediated TLR9 stimulation), further strengthens the fact that the initial defect in type I IFN production in IRF8^R294C^ is further amplified during the course of viral challenge. The observation of differential expression of *Ifnb* in pDCs due to a single point mutation *Irf8^R294C^*, coupled with the finding of impaired type I IFN production upon immune challenge in IRF8^R294C^, provides important insight in the role of IRF8 in the regulation of complex transcriptional program of DC development and function. We have demonstrated the inability of IRF8^R294C^ to induce interferon promoters, which might explain defective production of type I IFN in this phenotype. A previous study, which has indicated that IRF8 controls the chromatin access by regulating the stability of transcriptional complexes by recruitment of RNA polymerase II to IFN promoter region, extends support to our finding (52). The differential recruitment of RNA polymerase II by IRF8^R294C^ may have further nullified the type I IFN production by suppressing the feedback response of IFN, which is well established for production of more type I IFNs subsequently (52). How the point mutation IRF8^R294C^ changes the chromatin access and stability of the transcriptional machinery will be an important aspect of future research. IRF8 is well established to control the cDC1 and pDC lineage during developmental regime in precursor cells expressing IRF8 (1, 15). *Itgax-cre*-mediated deletion of *Irf8* identified that *Irf8* is important as a terminal selector of the cDC1 lineage but not of the monocyte or pDC lineage and suggested that IRF8 is critical to the functional gene modules in pDCs. The pDC subtype developed in *Irf8^fl/fl^Itgax-cre* mouse model was phenotypically distinct, having higher MHC II, CD11b, Ly6C, and CD11c expression and decreased expression of CD317 (43).

In the current study, for the first time, we report the defect in type I IFN production by IRF8^R294C^ mice upon viral challenge, casting great impact in deciphering the role of IRF8 in controlling developmental and functional paradigm of dendritic cells. It is intriguing that complete absence of IRF8 correlates to loss of pDC and cDC1 subsets, but IRF8^R294C^ mutation specifically blocks the developmental regime in cDC1. Here we have demonstrated that in spite of pDC development, the pDCs in IRF8^R294C^ mice tend to be functionally different than IRF8^WT^ pDCs. Further examinations in molecular level illustrating the mechanistic overview of the differential regulation of IRF8 in cDC1 and pDC will lead to unexplored arena of pDC and DC biology.

Once the IFN receptors are engaged after IFN production, they initiate downstream signaling cascade leading to generation of ISGs (14). Here we addressed the generation of ISGs upon NDV infection (or CpG-mediated TLR9 stimulation) in IRF8^WT^ and IRF8^R294C^ FLDCs and have found that absence of type I IFN production in IRF8^R294C^ further results in reduction of ISGs production, and this reduction in turn leads to immune suppression. However, in IRF8^R294C^ pDCs, studying the status of downstream signaling pathways such as JAK-STAT, which is known to mediate the production of ISGs upon activation by IFN receptor (14, 73), will provide further insight in this field. DCs, upon encountering immune challenges, upregulate the costimulatory molecules, which further helps in mediating adaptive immune response (13, 66). We explored the profile of pDC activation from IRF8^WT^ and IRF8^R294C^ FLDC cultures upon NDV infection (or CpG stimulation) and found that IRF8^R294C^ pDCs expressed a decreased level of costimulatory molecules such as CD40, CD86, and CD80 in comparison with IRF8^WT^, suggesting the impairment in pDC activation in IRF8^R294C^ mice. Further corroboration of our studies from *in vivo* model demonstrates the defect in type I IFN production and pDC activation in IRF8^R294C^ mice upon NDV infection. This defect in pDC activation may explain further exaggeration to increased NDV burden in IRF8^R294C^ mice. However, the underlying mechanism leading to the dearth of pDC activation requires additional characterization.

In conclusion, our study demonstrates IRF8 as a critical determinant of pDC functionality as the mutation *Irf8^R294C^* in mice does not affect pDC development but severely compromises the type I IFN-mediated crucial functions implemented by pDCs in response to viral pathogen. The consequences of *Irf8^R294C^* mutation are defect in three central aspects of innate immune response: (1) type I IFN production, (2) production of ISGs, and (3) pDC activation. These inadequacies in indispensable compartments of innate immune response may culminate to insufficient activation of adaptive immune response, leading to susceptibility of IRF8^R294C^ mice. Finally, IRF8^R294C^ mutant mice thus can be used as a model to elucidate the pDC functionality for designing therapeutic strategies of several autoimmune diseases where pDC dysregulation is associated with worse prognosis. Also, this mice model can help us in understanding the role of type I IFN production during viral infections.

## Data Availability Statement

The data presented in the study are deposited in the GEO repository, accession number GSE186051.

## Ethics Statement

The animal study was reviewed and approved by the Institute Animal Ethics Committee (IAEC) at National Institute of Immunology.

## Author Contributions

AD and PT contributed to study design, data analysis, and data interpretation. AD and KC have performed the experiments. AD, KC, and PT have contributed to manuscript preparation. HK helped in NDV infection studies. All authors contributed to the article and approved the submitted version.

## Funding

The authors would like to acknowledge financial support received from NII Core fund. KC was supported by fellowship from ICMR, and work was partially supported by grant #SERB/2016/005634.

## Conflict of Interest

The authors declare that the research was conducted in the absence of any commercial or financial relationships that could be construed as a potential conflict of interest.

## Publisher’s Note

All claims expressed in this article are solely those of the authors and do not necessarily represent those of their affiliated organizations, or those of the publisher, the editors and the reviewers. Any product that may be evaluated in this article, or claim that may be made by its manufacturer, is not guaranteed or endorsed by the publisher.
