# Supplementary material for: Mutation in Irf8 Gene (Irf8R294C ) Impairs Type I IFN-Mediated Antiviral Immune Response by Murine pDCs
Source: Front Immunol. 2021 Nov 17;12:758190. doi: 10.3389/fimmu.2021.758190 (PMC8635750; doi:10.3389/fimmu.2021.758190)
Supplement: Supplementary file 1 [file DataSheet_1.pdf]

Supplementary Figure 1

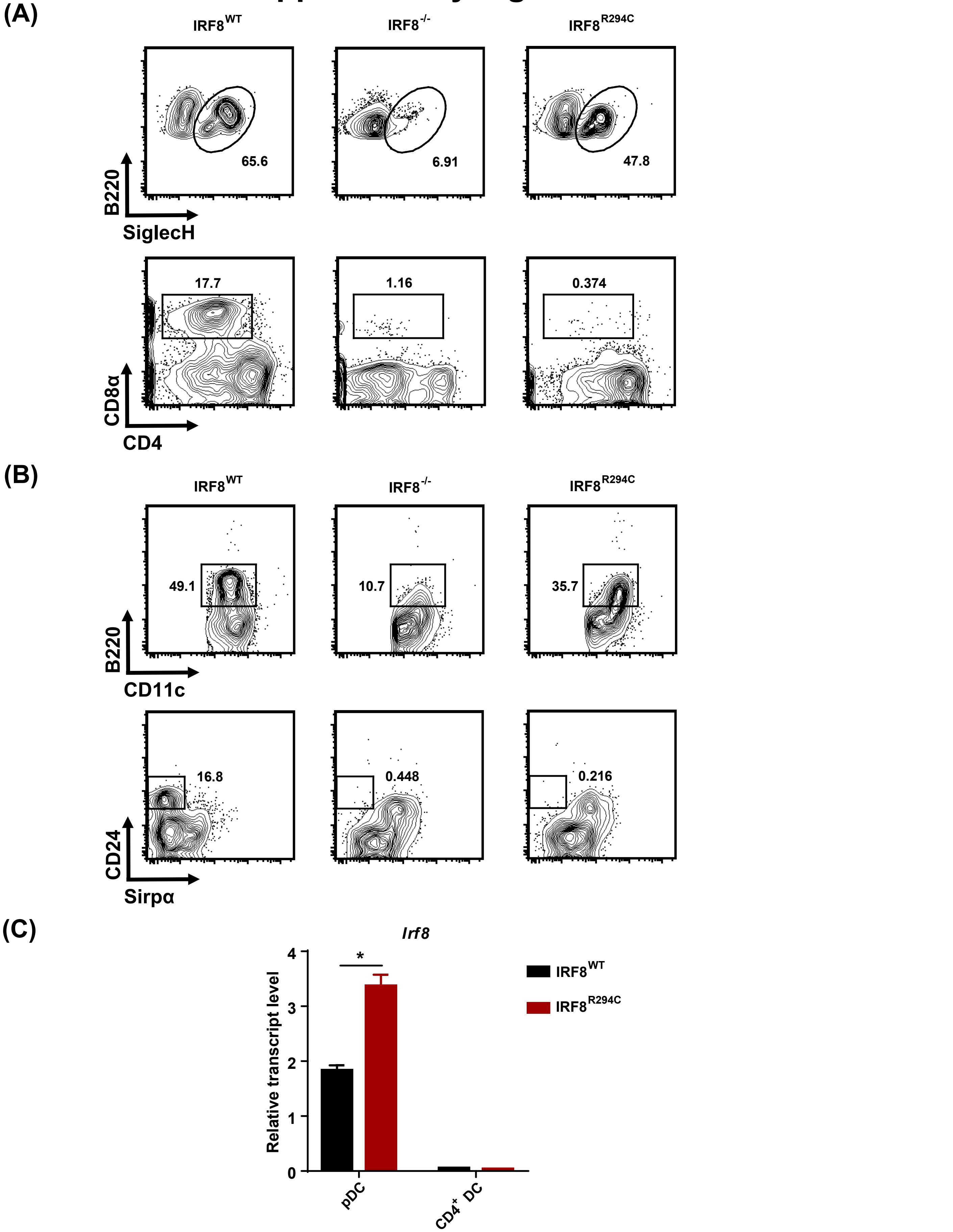

Supplementary figure 1. ***Irf8^{R294C}*** **mutation selectively abrogates cDC1 development. (A)** Flowcytometric analysis of splenic DC subsets depicts the absence of CD11c$^+$B220$^+$SiglecH$^+$ pDCs (analysed in live and CD11c$^+$B220$^+$gate) in IRF8$^{-/-}$ and CD11c$^+$B220$^-$CD8α$^+$CD4$^-$ cDC1s (analysed in live and CD11c$^+$B220$^-$gate) in both IRF8$^{-/-}$and IRF8^{R294C} mice.
(B) Characterisation of FLDC cultures exhibit the absence of CD11c$^+$B220$^+$ pDCs (analysed in live and CD11c$^+$gate) in IRF8$^{-/-}$ and abrogation of CD11c$^+$B220$^-$CD24$^+$Sirpα$^-$ cDC1s (analysed in live and CD11c$^+$B220$^-$gate) in both IRF8$^{-/-}$and IRF8^{R294C} mice. **(C)** qRT-PCR analysis of *Irf8* transcript in splenic CD11c$^+$B220$^+$ pDCs and CD11c$^+$B220$^-$CD8α$^-$CD4$^+$ DCs (CD4$^+$ DC) show increased level of *Irf8* transcript in IRF8^{R294C} pDCs in comparison to IRF8^{WT}. Data is representative of two independent experiments with error bar representing + SEM and *p<0.05. p value obtained from Student's t test. Data **(A,B)** are representative of three independent experiments.
