# Supplementary material for: Mutation in Irf8 Gene (Irf8R294C ) Impairs Type I IFN-Mediated Antiviral Immune Response by Murine pDCs
Source: Front Immunol. 2021 Nov 17;12:758190. doi: 10.3389/fimmu.2021.758190 (PMC8635750; doi:10.3389/fimmu.2021.758190)
Supplement: Supplementary file 2 [file DataSheet_2.pdf]

Supplementary Figure 2

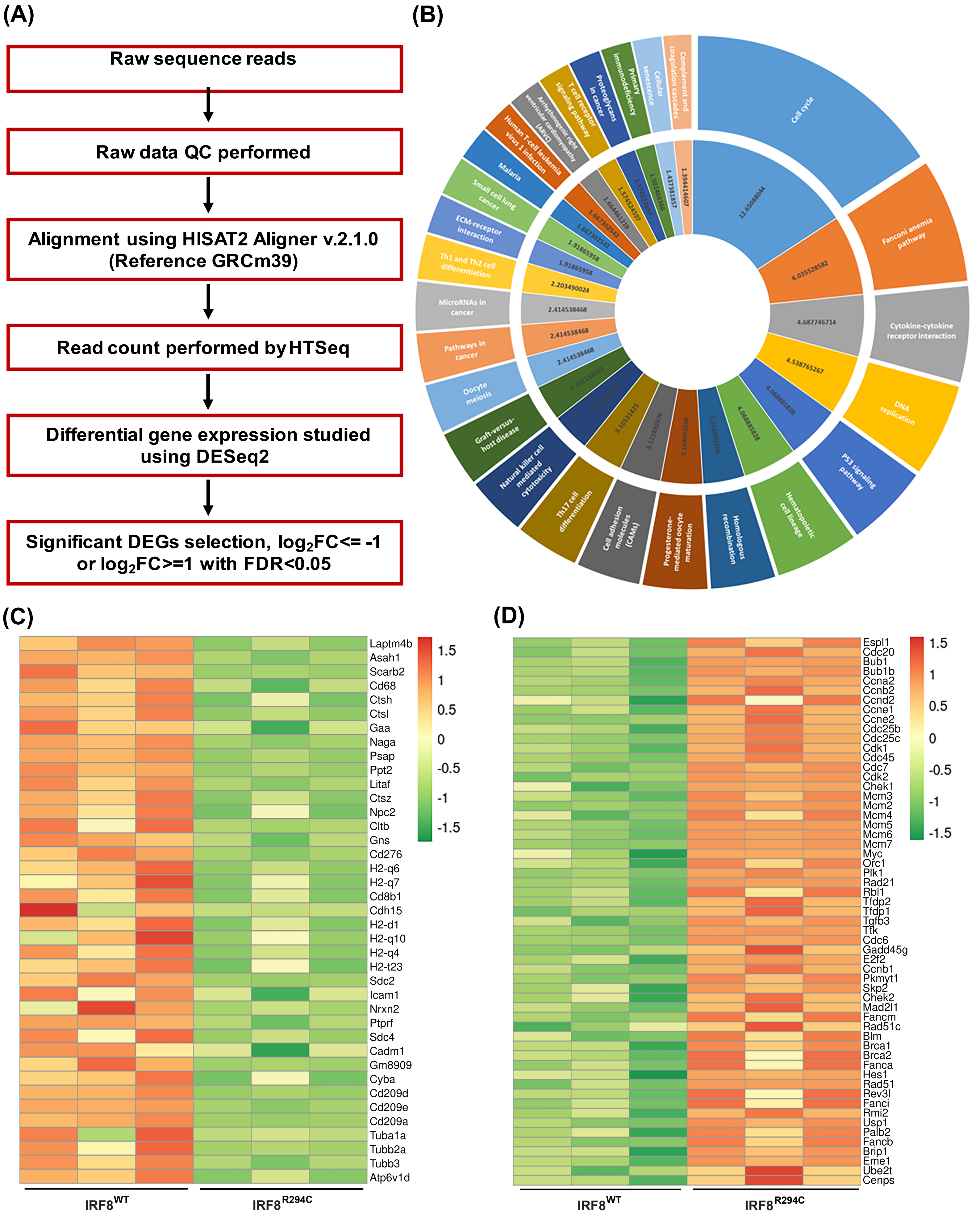

Supplementary figure 2. *Irf8^{R294C}* mutation leads to changes in transcriptome of pDC. (A) Schematic depicting workflow for RNA seq data analysis. **(B)** KEGG pathway analysis of downregulated genes in IRF8^{WT}, outer circle represents top downregulated pathways in IRF8^{WT} and inner circle indicates corresponding $-\log_{10}$FDR value of respective KEGG pathway. Heatmaps depicting **(C)** upregulated and **(D)** downregulated genes in IRF8^{WT} pDCs enriched in KEGG pathway with red and green denoting up and downregulation respectively.
