# Supplementary material for: Mutation in Irf8 Gene (Irf8R294C ) Impairs Type I IFN-Mediated Antiviral Immune Response by Murine pDCs
Source: Front Immunol. 2021 Nov 17;12:758190. doi: 10.3389/fimmu.2021.758190 (PMC8635750; doi:10.3389/fimmu.2021.758190)
Supplement: Supplementary file 3 [file DataSheet_3.pdf]

Supplementary Figure 3

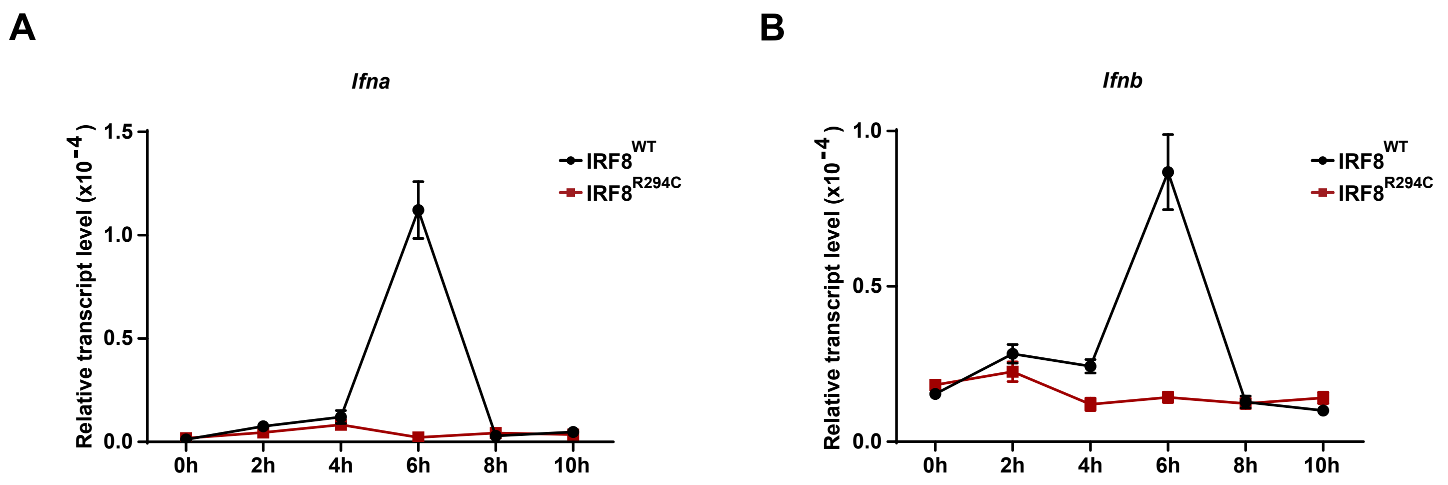

Supplementary figure 3. *Irf8*^{R294C} mutation impairs type I IFN production upon NDV infection in HEK293T cells. HEK293T cells stably expressing IRF8^{WT} or IRF8^{R294C} separately were infected with NDV for indicated time points and transcript levels of **(A)** *Ifna* and **(B)** *Ifnb* were analysed through qRT-PCR. Data are representative of three independent experiments with error bar representing ± standard error of mean (SEM).
