# Supplementary material for: Mutation in Irf8 Gene (Irf8R294C ) Impairs Type I IFN-Mediated Antiviral Immune Response by Murine pDCs
Source: Front Immunol. 2021 Nov 17;12:758190. doi: 10.3389/fimmu.2021.758190 (PMC8635750; doi:10.3389/fimmu.2021.758190)
Supplement: Supplementary file 4 [file DataSheet_4.pdf]

Supplementary Figure 4

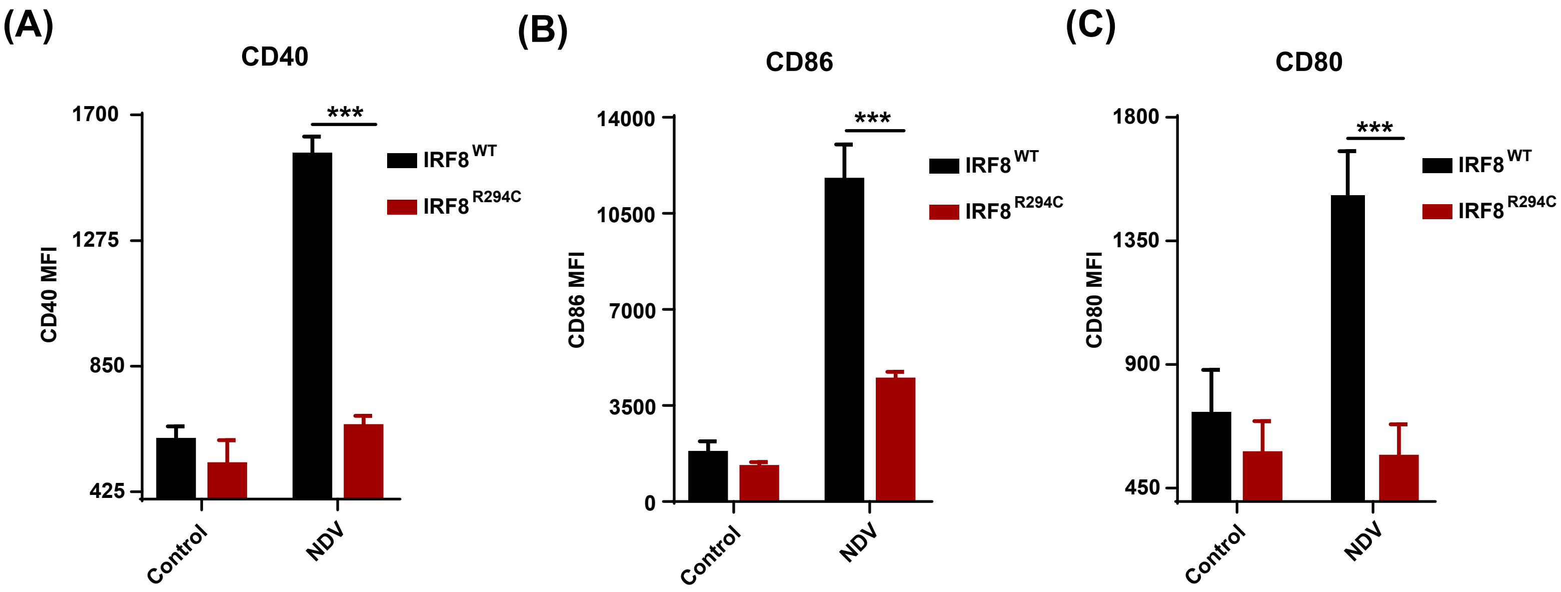

Supplementary figure 4. Splenic pDCs from IRF8^{R294C} mice are defective in upregulation of costimulatory molecules. Quantitation of MFI of **(A)** CD40, **(B)** CD86 and **(C)** CD80 in splenic CD11c^{+}SiglecH^{+} pDCs from control or NDV infected IRF8^{WT} and IRF8^{R294C} mice as described in Figure 6. Data (A-C) shown are mean value with error bar representing + SEM and *** p< 0.001 (n= 6 mice per group).
