# Supplementary material for: Mutation in Irf8 Gene (Irf8R294C ) Impairs Type I IFN-Mediated Antiviral Immune Response by Murine pDCs
Source: Front Immunol. 2021 Nov 17;12:758190. doi: 10.3389/fimmu.2021.758190 (PMC8635750; doi:10.3389/fimmu.2021.758190)
Supplement: Supplementary file 5 [file Table_1.pdf]

Supplementary table-1

Gene	Primer sequence (5'- 3')	Gene	Primer sequence (5'- 3')
Irf8	5'-AGGTGACCCGGAAGCTGTTT-3'<br>5'-CTTGCGGTTGCTGTGCAGTA-3'	***Oas1g***	5'-CTGGGTCATGGTAGTATCAAT-3'<br>5'-CCAGGGAGGTACATTCCCA-3'
Gapdh	5'-GTGTTCCTACCCCCAATGT-3'<br>5'-TGTCATCATACTTGGCAGGTTTC-3'	***Oas1a***	5'-CTGGGTCATGTTAATACTTCC-3'<br>5'-CCAGGGAGGTACATTCCTC-3'
Ifna	5'-CCTGAGARAGAAGAAACACAG-3'<br>5'-CTCTCCAGAYTTCTGCTCTG-3'	***Oasl2***	5'-GCCCCAGAGGGACTGGAA-3'<br>5'-CCCGAAAGAACCTCTCGATTCT-3'
Ifnb	5'-GCTCCTGGAGCAGCTGAATG -3'<br>5'-CGTCATCTCCATAGGGATCTTGA-3'	***Ifit3***	5'-AGGATGGCAGAACTGAGACGAT-3'<br>5'-TTCGCCTCCTCTGAAGAGTCTT-3'
Irf7	5'-GAAGAGGCTGGAAGACCAACTTC-3'<br>5'-GAATTGTCTTGGCGCAAGATAA-3'	***Oas2***	5'-ATGTCCTTCCCGCCTATGATG-3'<br>5'-CGGGCCTGAGGGTGAAGT-3'
Oas3	5'-CAGGCCCTTGAAGACAACAAAG-3'<br>5'-TTGGAAGGTTTTTGGCTCCTT-3'	***NDV***	5'-GGAGGATGTTGGCAGCATT-3'<br>5'-GTCAACATATACACCTCATC-3'

Supplementary table-1: List of primers used for qRT-PCR.
